# Typhoid Fever: The First Prescription

**Published:** 1883-01

**Authors:** P. P. Wells


### TYPHOID FEVER: THE FIRST PRESCRIPTION.

The first duty of the physician in attempting the treatment of this disease is a THOROUGH EXAMINATION OF ALL the elements of his case. This cannot be too strongly insisted on or too carefully performed. In no other disease is this so important, though it is indifferent in none. Having honestly discharged this duty and ascertained not only that he has the fever to combat, but *all* the elements it now reveals, let him consider them well and carefully before he decides on his first prescription, and be *positively sure* that he is *right* before he ventures on the first dose, for no subsequent effort in the case is of equal importance. If the first prescription be wrong, no subsequent pains may be sufficient to remedy the consequences of the blunder. A confusion of the case from this source has often been realized which no skill could remove. If the *first* prescription be right, the subsequent course is comparatively easy. If wrong, there is only vexation, difficulty, and anxiety before the physician; and to the patient and his friends it is too likely there is only a certain fearful looking for of pain, danger, and death. Let it never be forgotten that *time* is here of *no account,* if the question be of time or a wrong prescription. Let whatever of time the case may require for accuracy be given it, no matter to what extent, for it is infinitely better for the patient that we do nothing than that we do wrong. When the younger Bœnninghausen won his great and perhaps unparalleled success in the treatment of this fever with a single remedy, he recognized and practiced this principle. He made sure of the accuracy of his *first* prescription, and then he fully recognized and obeyed the second essential rule of successful practice, viz.: Having found the right remedy, keep to it—*change it for no other*—

but for the strongest reason. Let no impatience for more speedy results, nor that honest and honorable desire to do better which all right-minded practitioners must feel, and which is likely to be especially active with the young, tempt to do wrong by an unwise change of remedy. If the first be right, to change it for another will most likely be to give one less appropriate, and the result will too often cause bitter regret. The writer was years learning the importance of this rule of practice, and the memory of the experiences from which it was derived is mixed with much that is painful, which he has now no doubt a better practice might have avoided.

In treating typhoid fever it should be constantly borne in mind that it is not often that the disease can be crushed at the first blow. It may require patient and protracted watching and care before the looked-for amendment appears, but however long these may have been, the *time* furnishes no reason for substituting another remedy for that in use, this having been selected because its pathogenesis was most similar to the characteristic elements of the case. And now, whatever the time of its administration may have been, it is only to give place to a successor for a similar reason, greater resemblance to the elements of the case. Examples of both acute and chronic diseases are constantly met in practice which require medication for a longer or shorter period, according to the nature of the given case, before amendment becomes apparent, though the prescription may have been most accurate. Such cases are more frequent among typhoid fevers than other acute diseases. They call for the most careful revision of prescriptions, but never for a change of remedy except for the reason given above. Success in such cases only follows a steady adherence to that which has thus been ascertained to be right. When, as sometimes happens in this fever, this period of what may be termed latent medication is protracted days or even weeks, the course recommended requires more than common firmness and the exhibition of much of that quality called *nerve.* There can be no doubt but he who has not these qualities, or is unwilling to use them, is not fit for the responsible duty of treating them.—*Extract from Dr. P. P. Wells on Typhoid Fever in Am. Hom. Review.*

---

We are not to alternate remedies; we are to avoid giving Aconite in the first stage, with very rare exceptions (no one but a madman would think of giving it in a subsequent stage), and we are neither to give nor alternate Bryonia and Rhus simply because it is "typhoid fever."

